# Editorial for the Special Issue on Advanced Manufacturing Technology and Systems

**DOI:** 10.3390/mi14030495

**Published:** 2023-02-21

**Authors:** Youqiang Xing, Xiuqing Hao, Duanzhi Duan

**Affiliations:** 1 School of Mechanical Engineering, Southeast University, Nanjing 211189, China; 2 College of Mechanical and Electrical Engineering, Nanjing University of Aeronautics and Astronautics, Nanjing 210016, China; 3 School of Mechanical Engineering, Xi’an Jiaotong University, Xi’an 710049, China

Advanced manufacturing technology and systems (AMTSs) combine the principles of mechanical engineering with design innovation to create products and processes that are better, faster and more precise [1]. The core of AMTS is the design, fabrication and application of original and effective solutions related to manufacturing machines, process integration and systems to keep up with the dynamic needs of today’s ever-evolving industries [2].

Advanced manufacturing technology and systems cover a broad scope, involving manufacturing processes, machine tool design, system optimization, smart and flexible manufacturing, theoretical study and metrology [3,4]. From a fabrication point of view, AMTSs include physical, chemical, micro-/nanofabrication, machining and forming technology, additive manufacturing, non-traditional manufacturing processes, etc. From a system point of view, AMTSs include intelligent control, energy conversion for systems, optimization algorithms, smart sensors, etc. To improve the yield for mass productions, some effective testing and optimization methods are widely used.

This Special Issue comprises 19 original papers concerning recent advances in the research and development of AMTSs. Specifically, many research fields are covered as follows: machining processes (six papers), micro-/nano precision fabrication (four papers), system optimization (eight papers) and a review (one paper). These typical studies reveal the recent advances in advanced manufacturing, which are briefly summarized as follows.

Zhang et al. [5] studied the laser machining of SiC ceramics by ablation and polishing through the use of an infrared femtosecond laser, the laser ablation threshold of SiC ceramics was calculated, and the influence of pulse energy and the defocus amount of the femtosecond laser polishing of SiC ceramics were investigated. The optimal machining process parameters were obtained. Cheng et al. [6] analyzed the interfacial interaction mechanisms between a 4H-SiC wafer surface and diamond indenter during nanoscale scratching using distilled water without using an acid–base etching solution. They reported that the reaction between water and SiC on the wafer surface could be controlled. Microcracks can be avoided, and damage-free thinning of SiC wafers can be achieved by controlling the SiC–water reaction. In order to reduce the grinding force and enhance the removal rate when grinding zirconia ceramics, a nanosecond laser is used to ablate and grind the surface of a zirconia ceramic in a study conducted by Pang et al. [7]. Compared to the grinding surface without a laser-structure, a damage-free grinding surface was obtained through laser assistance. Wang et al. [8] established an ultrasonic elliptical vibration cutting (UEVC) finite element simulation model and UEVC cutting trajectory. A mechanism for the micro removal of materials in the UEVC process was obtained. Wang et al. [9] developed a water-dissolution polishing method to obtain near-damage-free KDP surfaces. They proved that the wetting characteristics of the polishing fluid should be improved during the optimization process of polishing fluid composition when using oil-based polishing fluids. Gao et al. [10] reviewed the current state of research that characterizes, estimated the effects of the process parameters on the mechanical properties, and summarized existing works on Fused Deposition Modeling (FDM). Zhang et al. [11] used atomic layer deposition (ALD) to regulate the line width of the one-dimensional grating standards with a pitch of 1000 nm, fabricated by electron beam lithography (EBL). They proved that the width of a single grating line in the standard could be regulated with great uniformity by precisely utilizing ALD. Meng et al. [12] proposed a side ohmic contact mode for the double-channel GaN/AlGaN epitaxial layer. Rectangle transmission line model (TLM) electrodes are prepared, and the specific contact resistance is tested at annealing temperatures ranging from 700 °C to 850 °C. Two sandwiched ZnO/Metal/ZnO transparent conductive thin films were deposited using magnetron sputtering technology by Lin et al. [13]. They revealed that the introduction of the Ti layer is beneficial to the overall properties of a ZnO (Ti/Cu) thin film compared to a ZnO (Cu) thin film with the same metal layer thickness, and annealing can improve the performance of the film systems. Xu et al. [14] proposed a novel chemically enhanced shear dilatancy polishing method (C-SDP). They revealed that C-SDP technology was a novel ultra-precision machining method that could achieve great surface qualities and polishing efficiency for tungsten. Du et al. [15] proposed two methods for predicting milling stability based on the composite Cotes and Simpson’s 3/8 formulas. Yu et al. [16] proposed an efficient algorithm using Field Programmable Gate Array (FPGA)-accelerated You Only Look Once (YOLO) v3 based on an attention mechanism. Xie et al. [17] proposed a prognostic method based on a back-propagation neural network for corroded pipeline systems. The rationality and effectiveness of the proposed prediction models were verified. Zhou et al. [18] introduced a methodology to model, design, fabricate and optimize an air-coupled ultrasonic transducers matching system. They indicated that the self-developed air-coupled ultrasonic transducer has a 20% higher amplitude than the product available on the market. Liu et al. [19] presented an integrated piezoresistive normal force sensor. The surface micromachined normal force sensor was transferred to the readout circuit chip with a temporary stiction effect handling process. Li et al. [20] established a Multiphysics-field-coupled simulation model of electric, flow and temperature fields during the electrochemical machining (ECM) of the miniature bearing outer ring based on the gas–liquid two-phase turbulent flow model, which was important in improving the machining accuracy of the outer ring of the ECM miniature bearing. Zhao et al. [21] proposed and manufactured an all-optical fiber sensor system based on the parallel structure of gold-plated Fiber Bragg grating (FBG) and quartz FBG, which could simultaneously measure temperature and pressure. Additionally, they designed and fabricated a high-sensitivity optical fiber Mach–Zehnder refractive index sensor and an all-fiber temperature and refractive dual-parameter-sensing Michelson interferometer based on a waist-enlarged bitaper [22,23].
